# Ensemble Consensus-Guided Unsupervised Feature Selection to Identify Huntington’s Disease-Associated Genes

**DOI:** 10.3390/genes9070350

**Published:** 2018-07-12

**Authors:** Xia Guo, Xue Jiang, Jing Xu, Xiongwen Quan, Min Wu, Han Zhang

**Affiliations:** College of Computer and Control Engineering, Nankai University, Tianjin 300350, China; guoxia@mail.nankai.edu.cn (X.G.); jiangxue@mail.nankai.edu.cn (X.J.); xujing95@mail.nankai.edu.cn (J.X.); wumin@nankai.edu.cn (M.W.); zhanghan@nankai.edu.cn (H.Z.)

**Keywords:** ensemble consensus guided unsupervised feature selection, disease-associated genes, Huntington’s disease, RNA-Seq data

## Abstract

Due to the complexity of the pathological mechanisms of neurodegenerative diseases, traditional differentially-expressed gene selection methods cannot detect disease-associated genes accurately. Recent studies have shown that consensus-guided unsupervised feature selection (CGUFS) performs well in feature selection for identifying disease-associated genes. Since the random initialization of the feature selection matrix in CGUFS results in instability of the final disease-associated gene set, for the purposes of this study we proposed an ensemble method based on CGUFS—namely, ensemble consensus-guided unsupervised feature selection (ECGUFS) in order to further improve the accuracy of disease-associated genes and the stability of feature gene sets. We also proposed a bagging integration strategy to integrate the results of CGUFS. Lastly, we conducted experiments with Huntington’s disease RNA sequencing (RNA-Seq) data and obtained the final feature gene set, where we detected 287 disease-associated genes. Enrichment analysis on these genes has shown that postsynaptic density and the postsynaptic membrane, synapse, and cell junction are all affected during the disease’s progression. However, ECGUFS greatly improved the accuracy of disease-associated gene prediction and the stability of the disease-associated gene set. We conducted a classification of samples with labels based on the linear support vector machine with 10-fold cross-validation. The average accuracy is 0.9, which suggests the effectiveness of the feature gene set.

## 1. Introduction

Neurodegenerative diseases seriously affect human health and quality of life. It is reported that half of the population aged seventy and over suffer from Alzheimer’s disease [1]. Neurodegenerative diseases are usually the result of one or more genes combined with one or more environmental factors. They are a kind of chronic disease characterized with complex symptoms. Alzheimer’s disease (AD) [2,3], Parkinson’s disease (PD) [4], and Huntington’s disease (HD) are some of the most common neurodegenerative diseases. Due to the complexity of neurodegenerative diseases, there are still many unknown parts of molecular mechanisms. It has been shown that the pathogenic gene of HD is *IT15*, and although it is widely expressed in various tissues within the body, only the neurons of specific tissues are damaged. Additionally, HD results from the misfolding of the protein Htt, and the symptoms of this disease usually develop after the age of 40 [5,6]. Due to the complexity of these diseases [7], identifying disease-associated genes is helpful for revealing the pathogenesis of the diseases.

With the rapid development of high-throughput sequencing technologies, working to identify disease-associated genes using statistic-based and machine learning methods to deal with gene expression data is a valuable endeavor. There are mainly three kinds of methods which can be used to identify disease-associated genes. Firstly, there are statistic-based methods, including the *t*-test method [8] and the fold change (FC) rank product method [8], which select differentially-expressed genes according to the statistically significant *p*-value by comparing the gene expression between disease samples and normal samples. Because the interaction between genes is not considered in these methods, the results have low accuracy. Secondly, there are machine learning methods, such as the flexible non-negative matrix factorization method (FNMF) [9], which works by sorting the genes according to a $l_{2}$ -norm by using a disease-driven relative gene expression matrix, whereby you can then select the top-ranking genes as disease-associated genes. Due to the random initialization of FNMF, the final gene rankings are somewhat unstable, which may result in some noise. Thirdly, network-based methods [10,11], such as the multi-label propagation (LP) clustering algorithm [12], are used to detect disease-associated gene modules. However, LP only relies on the similarity between gene expression data and lacks a feature gene selection process, which makes the selected disease-associated gene set imprecise. Consequently, the above methods have some limitations in accurately identifying disease-associated genes to some extent.

Disease-associated gene identification can be seen as a feature selection problem in the machine learning field. Due to the sample labels being unknown and the acquisition of label information being costly in many cases [13], unsupervised machine learning methods are more promising when dealing with biological data [14]. Due to the importance of consensus-clustering in feature gene selection, we used the consensus-guided unsupervised feature selection (CGUFS) [15] method to identify disease-associated genes. The random initialization of the feature selection matrix in CGUFS results in instability of the final feature gene set. Ensemble methods have been used effectively in bioinformatics [16,17] in recent years. For example, ensemble classifiers are applied in Zou et al. [18] to improve tRNAscan-SE annotation results. Since Zou et al. [19] also uses ensemble support vector machines to detect *N*^6^–methyladenosine sites from RNA transcriptomes, we designed the ensemble strategy using bagging [20] to improve the accuracy of the disease-associated gene prediction.

Based on the above analysis, we proposed an ensemble method based on CGUFS, or namely, ensemble consensus-guided unsupervised feature selection (ECGUFS), to help identify disease-associated genes. Firstly, we used bootstrap aggregation to generate multiple bags from the RNA sequencing (RNA-Seq) data. For each bag, the gene weights and gene-ranked list were obtained. Secondly, the area under the receiver operating characteristic (ROC) curve of the ranked list was calculated to measure the accuracy of the disease-associated gene prediction. Finally, we obtained ensemble gene weights through a linear combination of all the gene weights. The genes were sorted in descending order according to these ensemble gene weights, and the higher the ranking, the more disease-associated was the gene. The experimental results showed that ECGUFS improved both the accuracy of the disease-associated gene prediction and the stability of the feature set. Compared with other methods for predicting disease-associated genes, ECGUFS has proved superior in the analysis of gene expression data for diseases with complex pathologies and also in the identification of disease-associated genes. Finally, we conducted a classification of disease samples from normal samples using the support vector machine. The experimental results further verified the effectiveness of the feature gene set.

## 2. Materials and Methods

In this section, we will first introduce the CGUFS method [15]; second, describe the evaluation criteria of disease-associated gene prediction accuracy; and finally, use a bagging integration strategy to integrate the results of CGUFS, or namely, ECGUFS, to obtain a more unified disease-associated gene set.

### 2.1. Consensus-Guided Unsupervised Feature Selection

Let $X={\left[x_{ij}\right]}_{g\times s}$ denote the gene expression matrix. $x_{ij}$ represents the expression level of gene i in sample j. A clustering result of s samples is represented by an indicator matrix $H\in {\left\{0,1\right\}}^{s\times K}$, where $h_{jk}=1$ denotes that sample j belongs to the k−th cluster, and K is the number of clusters. $H=\left\{H^{\left(1\right)},H^{\left(2\right)},\cdots H^{\left(r\right)}\right\}$ are the r basic clustering results of X in consensus clustering.

Part of CGUFS is the design of the following objective function, Equation (1). When the objective function is minimized to get the feature selection matrix Z and the consensus indicator matrix $H^{*}$, the $l_{2}$ -norm of each row of the feature selection matrix Z is used as the weight of each feature gene. In order to identify the disease-associated genes, the genes need to be sorted into descending order according to weight. The highly-ranked genes are the disease-associated genes.

(1) $$\underset{H^{*},G,Z}{\min}\;-\alpha \sum_{i=1}^{r}U_{c}(H^{*},H^{\left(i\right)})+{\left\|X^{T}Z-H^{*}G\right\|}_{F}^{2}+\beta {\left\|Z\right\|}_{2,1},$$

where $H^{*}$ is the consensus indicator matrix of the consensus clustering, $U_{c}$ is a function that measures the similarity of two basic clustering results to obtain the consensus clustering result [21]. $Z\in R^{g\times \;K}$ is the feature selection matrix, G is a mapping matrix between $X^{T}Z$ and $H^{*}$, and both α and β are parameters that control consensus clustering and sparse learning.

Specifically, CGUFS is an unsupervised feature selection method that does not require label information. As CGUFS adopts consensus clustering for pseudo-label learning, it can greatly improve clustering accuracy. CGUFS performs sparse regularization constraint on the feature selection matrix, which reduces the model’s complexity and improves its operation rate. The optimization solution of the objective function is as follows [15]:

Firstly, when given Z, update $H^{*}$ and G. A part of Equation (1) can be converted to Equation (2) in order to simplify the optimization process [22]:

(2) $$\sum_{i=1}^{r}U_{c}(H^{*},H^{\left(i\right)})=-{\left\|B-H^{*}C\right\|}_{F}^{2}+cons\tan t,$$

where $B=[H^{\left(1\right)},H^{\left(2\right)},\cdots H^{\left(r\right)}]$ is a matrix of the r basic clustering results of consensus clustering, and $C=[C^{\left(1\right)},C^{\left(2\right)},\cdots C^{\left(r\right)}]$ is the center of B. Thus, $H^{*}$ and G can be updated through the optimization of Equation (3).

(3) $$\underset{H^{*},G,C,Z}{\min}\;\alpha {\left\|B-H^{*}C\right\|}_{F}^{2}+{\left\|X^{T}Z-H^{*}G\right\|}_{F}^{2}.$$

Secondly, an optimization approach similar to *K*-means clustering is used to update $H^{*}$ and G [15]. Let $U=[\sqrt{\alpha }B{\;X}^{T}Z]$, where $u_{l}$ is the i−th row of U. Update $H^{*}$, G, and C in the following way.

(4) $$\begin{array}{c} \underset{H^{*},C,G,Z}{\min}\;\alpha {\left\|B-H^{*}C\right\|}_{F}^{2}+{\left\|X^{T}Z-H^{*}G\right\|}_{F}^{2}\\ \Leftrightarrow \underset{H^{*}}{\min}\sum_{k=1}^{K}\sum_{u_{l}\in C_{k}}f(u_{l},m_{k}) \end{array},$$

where $m_{k}$ is the k−th center of matrix U, and G is the last K row of center U.

Finally, update Z by giving $H^{*}$ and G. Since Z only appears in the last two terms of Equation (1), update Z by optimizing the last two items. Let $L={\left\|X^{T}Z-H^{*}G\right\|}_{F}^{2}+\beta {\left\|Z\right\|}_{2,1}$, and let the derivative of L to Z be 0. The updated equation of Z is:

(5) $$Z={\left(XX^{T}+\beta F\right)}^{-1}XH^{*}G,$$

where $F=diag(\frac{1}{2{\left\|Z_{1\cdot }\right\|}_{2}},\cdots \frac{1}{2{\left\|Z_{g\cdot }\right\|}_{2}})$.

In our proposed method, the weight of the gene can be calculated by using Equation (6). $w_{i}$ represents the weight of gene i, and W represents a vector of the weights for all genes.

(6) $$w_{i}={\left\|Z_{i\cdot }\right\|}_{2},$$

(7) $$W=\left[w_{1},\cdots ,w_{g}\right].$$

### 2.2. Evaluation

We used the true positive rate (TPR), false positive rate (FPR), precision (P), and recall (R) to assess the accuracy of disease-associated gene prediction. The ROC curve was plotted using TPR and FPR, the precision-recall (PR) curve was plotted using P and R, and the area under the ROC curve (AUC) and the area under the PR curve (AUPR) were used as measures of prediction accuracy [23].

### 2.3. Ensemble Consensus-Guided Unsupervised Feature Selection

Since the random initialization of the feature selection matrix in CGUFS caused instability in the final gene ranking, this work proposed a bagging integration strategy to integrate the results of CGUFS, or namely, ECGUFS, to obtain a more unified disease-associated gene set and also to improve the accuracy of the final gene set.

Firstly, we used bootstrap aggregation to generate b bags from $X={\left[x_{ij}\right]}_{g\times s}$. Each bag had c samples, where c is generally equal to the number of samples. For the i−th bag, the gene weights were calculated based on CGUFS and denoted as $W_{i}$. The gene-ranked list was obtained through $W_{i}$. Secondly, we calculated the area under the ROC of the gene-ranked list, which is denoted by $a_{i}$. Finally, we used Equation (8) to calculate the ensemble gene weights.

(8) $$W_{final}=\sum_{i=1}^{b}a_{i}W_{i}.$$

The genes were sorted in descending order according to $W_{final}$. Highly-ranked genes indicated that they played an important role in the discrimination between disease samples and normal ones.

**Figure 1:**
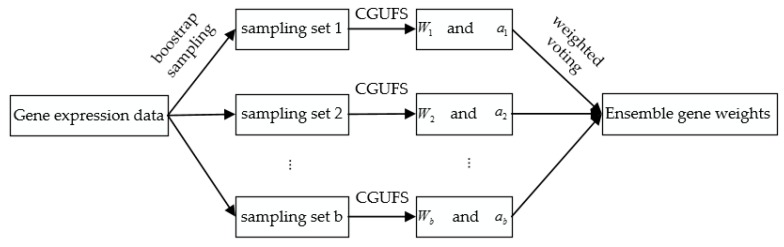
Flow chart of the ensemble consensus-guided unsupervised feature selection (ECGUFS) algorithm. Consensus-guided unsupervised feature selection (CGUFS); $W_{i}$: a vector of weights for all genes; $a_{i}$: the area under the receiver operating characteristic (ROC) curve of the gene-ranked list.

Figure 1 shows the flow chart of ECGUFS. After these processes, the final feature gene set is obtained.

The algorithm of ECGUFS can be described as follows in (Table 1).

## 3. Results

### 3.1. Gene Expression Data

The data used in this study was obtained from HDinHD (http://www.hdinhd.org), which is the gene expression data of HD mice obtained by RNA-Seq technology. The dataset contained mouse liver tissue, cortex tissue, and striatum tissue, and the genotypes of the mice were poly Q20, poly Q80, poly Q92, poly Q111, poly Q140, and poly Q175. There were 8 samples for each genotype [24]. The mice whose genotype was poly Q20 were normal mice, whereas mice with all other genotypes were diseased mice. The longer the poly Q, the heavier was the phenotype of the diseased mice. There were 23,351 genes in the dataset, and most of the calculation methods used for data analysis were based on differential expression genes to identify disease-associated genes. As it was difficult to identify the genes whose expression levels did not change during the disease’s progression, we preprocessed the gene expression data in order to reduce computational complexity. Firstly, the gene whose expression value was zero in any sample was deleted according to the $l_{0}$ -norm. Then, we normalized the gene expression data for each sample. We ranked the genes into descending order according to the gene variance in different samples, and only the top 4000 genes were selected. We then got the union set of 4000 genes in the three tissues and added the modifier gene set [24]. Finally, 6723 genes were selected from the entire genome. As it has been reported that striatum tissue can be seriously affected by the length of poly Q, we used striatum tissue data as experimental data in this study.

The modifier genes are from [24], including 520 genes, 89 of which are disease-associated genes and the rest of which are non-disease-associated genes. It should be noted that we normalized the gene expression data by each gene to ensure the convergence of the ECGUFS.

### 3.2. Parameter Setting

In ECGUFS, we set the number of clusters to K=6 as the number of mouse genotypes was six. We set the number of basic clustering results to r=100 to ensure the robustness of consensus clustering [15]. We set the parameter that controlled the consensus clustering to $\alpha =10^{4}$, and the parameter that controlled the sparse regularization to β=1. The higher α and β were, the better the performance of ECGUFS. When β was larger than 1, the results stabilized and were unaffected by α, according to [15]. The number of bags was set to b=20, and the number of samples in a bag to c=48.

### 3.3. Performance Comparison between Ensemble Consensus-Guided Unsupervised Feature Selection and Other Methods

To further verify the prediction accuracy of ECGUFS, we conducted comparative experiments using statistic-based methods, machine learning methods, and network-based methods on the above data set. The *t*-test method [8], FC [8], edgeR tool [25], limma [26], FNMF [9], the joint non-negative matrix factorization meta-analysis method (jNMFMA) [27], LP [12], and CGUFS [15] were used as comparison methods. For non-parametric methods, such as the *t*-test, FC, edgeR, limma, and LP, only one experiment was conducted. The experimental results of parametric methods, such as CGUFS, FNMF, jNMFMA, and ECGUFS were unstable due to the random initialization. Consequently, this work conducted 10 experiments for each parametric method. The mean and standard deviation of the prediction accuracy of the 10 experiments were calculated to evaluate the performance of the corresponding method.

The experimental results of FNMF, jNMFMA, CGUFS, and ECGUFS are shown in Table 2. From Table 2, we can see that the average AUC and AUPR of ECGUFS are better than FNMF, jNMFMA, and CGUFS, which shows that ECGUFS improved the accuracy of the disease-associated gene prediction. To analyze the performance of the nine methods in detail, a set of best-performing experiments were selected for further comparison.

**Figure 2:**
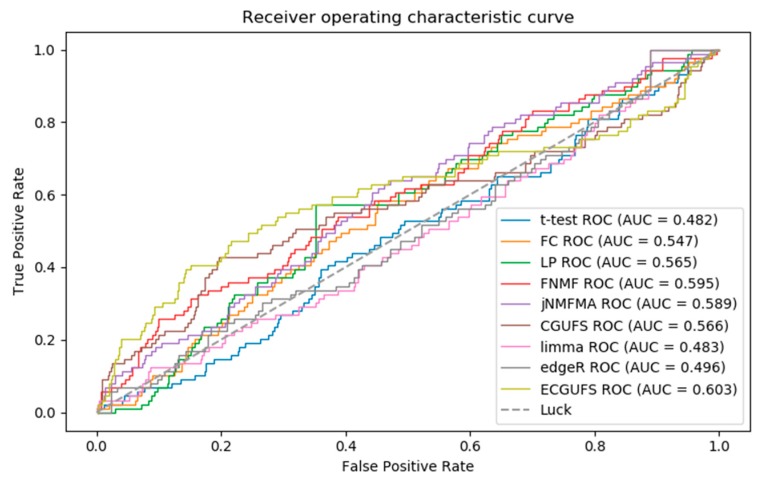
ROC curves of the *t*-test, fold change (FC), multi-label propagation clustering algorithm (LP), FNMF, jNMFMA, CGUFS, limma, edgeR, and ECGUFS prediction results.

Figure 2 shows the ROC curves for the prediction accuracy of the *t*-test, FC, edgeR, limma, LP, FNMF, jNMFMA, CGUFS, and ECGUFS. It can be seen from Figure 2 that the AUC of ECGUFS is better than the other eight methods. However, the above methods could not effectively distinguish the disease-associated genes from the non-disease-associated genes in the modifier gene set. Possible reasons for this may be that the expression levels of the disease-associated genes did not change significantly during the disease’s progression, or that a large number of gene expression levels had changed during the disease’s progression, thereby making it difficult to identify the disease-associated genes [28].

**Figure 3:**
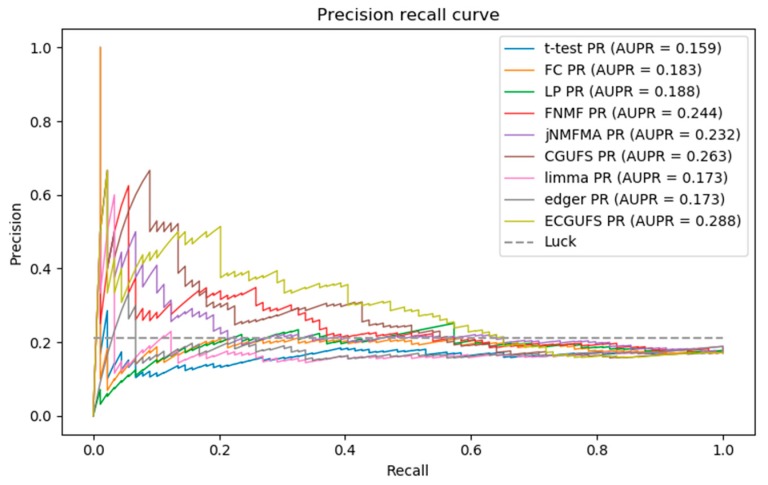
PR curves of the *t*-test, FC, LP, FNMF, jNMFMA, CGUFS, limma, edgeR, and ECGUFS prediction results.

Figure 3 shows the PR curves of the nine methods. It can be seen from Figure 3 that the AUPR of ECGUFS is better than the other eight methods. As ECGUFS has a higher prediction accuracy for highly-ranked genes, it indicates that ECGUFS can better distinguish disease-associated genes from non-disease-associated genes for highly-ranked genes.

Briefly, it can be seen from Table 2, and Figure 2 and Figure 3 that the performance of ECGUFS is better than CGUFS. The AUC and AURP standard deviation of the 10 experiments by ECGUFS is small, indicating the stability of ECGUFS. Experimental results show that ECGUFS is more suitable for identifying disease-associated genes than the other eight methods.

From Figure 3, we can see that when the recall rate is less than 0.2, ECGUFS has high prediction accuracy. Since there are 89 disease-associated genes in the modifier gene set, the top 18 (0.2×89) disease-associated genes have higher prediction accuracy. As the last of the 18 genes ranked roughly at about 1000 in the overall ranking, this work further calculates the coincidence degree of the top 1000 genes in the ranked lists of any two experiments. The results are shown in Table 3, where we can see that the coincidence degree is greater than 60%. The results also show that when ECGUFS is used to identify disease-associated genes, many overlapped genes can be identified under the condition of changes in sample size. Through the integration analysis we found that of the top 1000 genes, there were 287 overlapped ones of the ten experiment-ranked lists. In addition, we also calculated the coincidence degree of the top 2000 genes in different ranked lists. The results are shown in Table 4. There are 962 overlapped genes in the top 2000 genes. The high coincidence degree indicates that ECGUFS can improve the stability of a disease-associated gene set.

To verify the effectiveness of the overlapped 287 genes, we conducted a classification of disease samples from normal samples based on the support vector machine (SVM) [29], using ten-fold cross-validation. The average accuracy is 0.9, suggesting the effectiveness of the 287 feature genes.

### 3.4. Enrichment Analysis

We used the functional clustering tool DAVID [30] to perform enrichment analysis on 287 overlapped genes to further understand the functional annotation of these genes. Table 5 shows the functional annotation results. In the first clustering module, the cellular component (CC) annotations include postsynaptic density and the postsynaptic membrane, synapse, and cell junctions, indicating that the morphology of the cells has undergone major changes during the progression of HD. In fact, the connection between neurons of the striatum tissue gradually became sparse and the nerve cells slowly died during the progression of the disease [31,32]. In the second clustering module, the biological process (BP) annotations include a fatty acid metabolic process and a fatty acid biosynthetic process. The Molecular Function (MF) annotation includes transferase activity, transferring acyl groups other than amino-acyl groups. The Kyoto encyclopedia of genes and genomes (KEGG) pathway annotation includes fatty acid metabolism. In the third clustering module, the MF annotations include transferase activity, kinase activity, nucleotide binding, ATP (adenosine triphosphate) binding, protein kinase activity, and protein serine/threonine kinase activity. The BP annotations include phosphorylation and protein phosphorylation. In the fourth clustering module, the BP annotations include learning or memory, regulation of synaptic plasticity, and embryo development. Studies have shown that HD is related to mental disorders and cognitive decline. In the fifth clustering module, the MF annotation includes cadherin binding involved in cell–cell adhesion. The BP annotation includes cell–cell adhesion. The above molecular functions and biological processes are most likely to be affected during the disease’s progression. Studies have shown that Huntington’s disease is caused by excessive repetition of the CAG sequence of the huntingtin gene on the fourth chromosome. It leads to a misfolding of the Htt protein, which affects protein transport, gene regulation, and other biological processes. It eventually leads to sparse cell connections, neuronal apoptosis, and the formation of amyloid plaques in the striatum of the brain [33,34,35].

## 4. Discussion

In this work we proposed ECGUFS based on CGUFS. The main goal is that we proposed a bagging integration strategy to integrate the results of CGUFS. ECGUFS can effectively select disease-associated genes when the labels are unknown. Experimental results verify the better feasibility and stability of ECGUFS. In addition, we conducted an enrichment analysis of the overlapped 287 genes to further explore the molecular pathogenesis of HD, as well as to provide guidance for subsequent biological validation.

## Figures and Tables

**Table 1:** Ensemble consensus-guided unsupervised feature selection.

**Input:** X: The gene expression matrix; B: The matrix of r basic clustering results; α, β: Parameters; b: The number of bags; c: The number of samples in one bag. Initialize $W_{final}=0$; 1: For i=1,2,⋯b Initialize $H^{*}$,Z,F; 2: repeat; 3: build the matrix $U=[\sqrt{\alpha }B{\;X}^{T}Z]$; 4: run K-means on U to update $H^{*}$ and G; 5: update $Z={\left(XX^{T}+\beta F\right)}^{-1}XH^{*}G$; 6: update F; 7: until the value of the objective function remains unchanged. 8: Obtain the gene weights $W_{i}$ according to Equation (7); sort genes according to $W_{i}$ to get the gene-ranked list; 9: get the area under the ROC of the gene-ranked list $a_{i}$; 10: End 11: Calculate the ensemble gene weights according to Equation (8). $W_{final}+=a_{i}W_{i}$ 12: **Output**: $W_{final}$

Note: Initialize the elements of Z between 0 and 1, $F=diag(\frac{1}{2{\left\|Z_{1\cdot }\right\|}_{2}},\cdots \frac{1}{2{\left\|Z_{g\cdot }\right\|}_{2}})$. Initialize $H^{*}$ through consensus clustering.

**Table 2:** Performance mean ± standard deviation of FNMF, jNMFMA, CGUFS, and ECGUFS.

	FNMF	jNMFMA	CGUFS	ECGUFS
AUC	56.0 ± 1.9	56.7 ± 1.6	54.3 ± 1.5	59.2 ± 0.8
AUPR	20.4 ± 1.9	20.7 ± 1.6	22.5 ± 1.8	29.4 ± 1.9

FNMF: Flexible non-negative matrix factorization method; jNMFMA: Joint non-negative matrix factorization meta-analysis method; AUC: Area under the ROC curve; AUPR: Area under the precision-recall (PR) curve.

**Table 3:** The number of overlapped genes between the top 1000 genes of any two ranked lists obtained by ECGUFS.

	E2	E3	E4	E5	E6	E7	E8	E9	E10
E1	710 (71%)	705 (70.5%)	686 (68.6%)	677 (67.7%)	695 (69.5%)	679 (67.9%)	663 (66.3%)	691 (69.1%)	666 (66.6%)
E2		697 (69.7%)	686 (68.6%)	657 (65.7%)	686 (68.6%)	721 (72.1%)	676 (67.6%)	737 (73.7%)	682 (68.2%)
E3			689 (68.9%)	677 (67.7%)	691 (69.1%)	683 (68.3%)	655 (65.5%)	678 (67.8%)	665 (66.5%)
E4				684 (68.4%)	704 (70.4%)	696 (69.6%)	681 (68.1%)	715 (71.5%)	668 (66.8%)
E5					659 (65.9%)	657 (65.7%)	674 (67.4%)	665 (66.5%)	664 (66.4%)
E6						670 (67.0%)	670 (67.0%)	691 (69.1%)	690 (69.0%)
E7							666 (66.6%)	707 (70.7%)	669 (66.9%)
E8								678 (67.8%)	649 (64.9%)
E9									682 (68.2%)

Note: E1 represents experiment 1 using ECGUFS.

**Table 4:** The number of overlapped genes between the top 2000 genes of any two ranked lists obtained by ECGUFS.

	E2	E3	E4	E5	E6	E7	E8	E9	E10
E1	1593 (79.7%)	1598 (79.9%)	1565 (78.3%)	1570 (78.5%)	1603 (80.2%)	1569 (78.5%)	1547 (77.4%)	1589 (79.5%)	1564 (78.2%)
E2		1623 (69.7%)	1589 (79.5%)	1550 (77.5%)	1621 (81.1%)	1618 (80.9%)	1534 (76.7%)	1621 (81.1%)	1595 (79.8%)
E3			1582 (79.1%)	1589 (79.5%)	1610 (80.5%)	1603 (80.2%)	1559 (78.0%)	1599 (80.0%)	1590 (79.5%)
E4				1573 (78.7%)	1605 (80.3%)	1563 (78.2%)	1570 (78.5%)	1592 (79.6%)	1567 (78.4%)
E5					1569 (78.5%)	1545 (77.3%)	1575 (78.8%)	1572 (78.6%)	1567 (78.4%)
E6						1597 (79.9%)	1570 (78.5%)	1607 (80.4%)	1619 (81.0%)
E7							1557 (77.9%)	1615 (80.8%)	1584 (79.2%)
E8								1561 (78.1%)	1550 (77.5%)
E9									1596 (79.8%)

**Table 5:** The functional annotation clusterings of the 287 overlapped genes.

Annotation Cluster	Category	Annotation	Count	*p* Value	Benjamini
1 Enrichment Score: 3.02	GOTERM_CC_DIRECT	Postsynaptic density	16	4.2 × 10^−7^	1.3 × 10^−4^
	GOTERM_CC_DIRECT	Postsynaptic membrane	10	2.2 × 10^−3^	9.1 × 10^−2^
	GOTERM_CC_DIRECT	Synapse	14	1.3 × 10^−2^	2.4 × 10^−1^
	GOTERM_CC_DIRECT	Cell junction	15	7.4 × 10^−2^	4.9 × 10^−1^
2 Enrichment Score: 1.93	GOTERM_BP_DIRECT	Fatty acid metabolic process	9	9.3 × 10^−4^	4.8 × 10^−1^
	GOTERM_BP_DIRECT	Fatty acid biosynthetic process	5	1.5 × 10^−2^	8.4 × 10^−1^
	KEGG_PATHWAY	Fatty acid metabolism	4	3.6 × 10^−2^	8.7 × 10^−1^
	GOTERM_MF_DIRECT	Transferase activity, transferring acyl groups other than amino-acyl groups	3	3.7 × 10^−2^	7.6 × 10^−1^
3 Enrichment Score: 1.81	GOTERM_MF_DIRECT	Transferase activity	37	2.4 × 10^−4^	1.1 × 10^−1^
	GOTERM_BP_DIRECT	Phosphorylation	17	5.7 × 10^−3^	6.4 × 10^−1^
	GOTERM_MF_DIRECT	kinase activity	18	8.5 × 10^−3^	5.7 × 10^−1^
	GOTERM_MF_DIRECT	Nucleotide binding	38	1.4 × 10^−2^	6.3 × 10^−1^
	GOTERM_MF_DIRECT	ATP binding	30	2.6 × 10^−2^	7.3 × 10^−1^
	GOTERM_BP_DIRECT	Protein phosphorylation	14	3.5 × 10^−2^	9.6 × 10^−1^
	GOTERM_MF_DIRECT	Protein kinase activity	12	9.6 × 10^−2^	8.6 × 10^−1^
	GOTERM_MF_DIRECT	Protein serine/threonine kinase activity	9	2.1 × 10^−1^	9.4 × 10^−1^
4 Enrichment Score: 1.58	GOTERM_BP_DIRECT	Learning or memory	5	4.1 × 10^−3^	6.9 × 10^−1^
	GOTERM_BP_DIRECT	Regulation of synaptic plasticity	4	1.7 × 10^−2^	8.4 × 10^−1^
	GOTERM_BP_DIRECT	Embryo development	3	2.7 × 10^−1^	9.9 × 10^−1^
5 Enrichment Score: 1.40	GOTERM_CC_DIRECT	Cell–cell adherens junction	10	2.0 × 10^−2^	2.9 × 10^−1^
	GOTERM_MF_DIRECT	Cadherin binding involved in cell–cell adhesion	9	3.3 × 10^−2^	7.7 × 10^−1^
	GOTERM_BP_DIRECT	Cell­–cell adhesion	6	9.7 × 10^−2^	9.9 × 10^−1^
